# Supplementary material for: Optimization of Biocompatibility for a Hydrophilic Biological Molecule Encapsulation System
Source: Molecules. 2022 Feb 27;27(5):1572. doi: 10.3390/molecules27051572 (PMC8911823; doi:10.3390/molecules27051572)
Supplement: Supplementary file 1 [file molecules-27-01572-s001.zip › molecules-1466737-supplementary.pdf]

Supplemental Online Materials

Supplemental Discussion

Pearson correlation analyses of formulations prepared in each solvent are presented according to preparation method in Supplemental Figure S1. It should be noted that the smaller size of these data subsets (324 data points in each) limit the statistical value of the observed correlations. Comparison of these analyses, however, reveals common trends and differences between solvent-specific data sets that are worth noting. For most solvents, the observed trends are comparable regardless of sample preparation method. The phase-inversion temperature (PIT) preparation method is substantially more labor-intensive and time-consuming than the solvent-displacement approach. The PIT method also involves high temperatures that would likely limit applicability to protein-based cargo for which high temperatures can result in denaturation. Solvent-specific trends are difficult to unambiguously interpret for the highly toxic solvents (Transcutol HP, Capmul MCM, Capryol 90, Iso-octane, and Captex 355). For low-toxicity solvents (Lauroglycol 90, Lauroglycol FCC, and Labrafac PG), the observed pairwise trends will inform future development of mixtures involving the 10MAG/LDAO ratios in these and in other solvents of similar hydrophobicity. For example, the correlations between surfactant molarity and toxicity vary considerably between these solvents, thus these data indicate that this parameter will need to be optimized for each solvent in future testing.

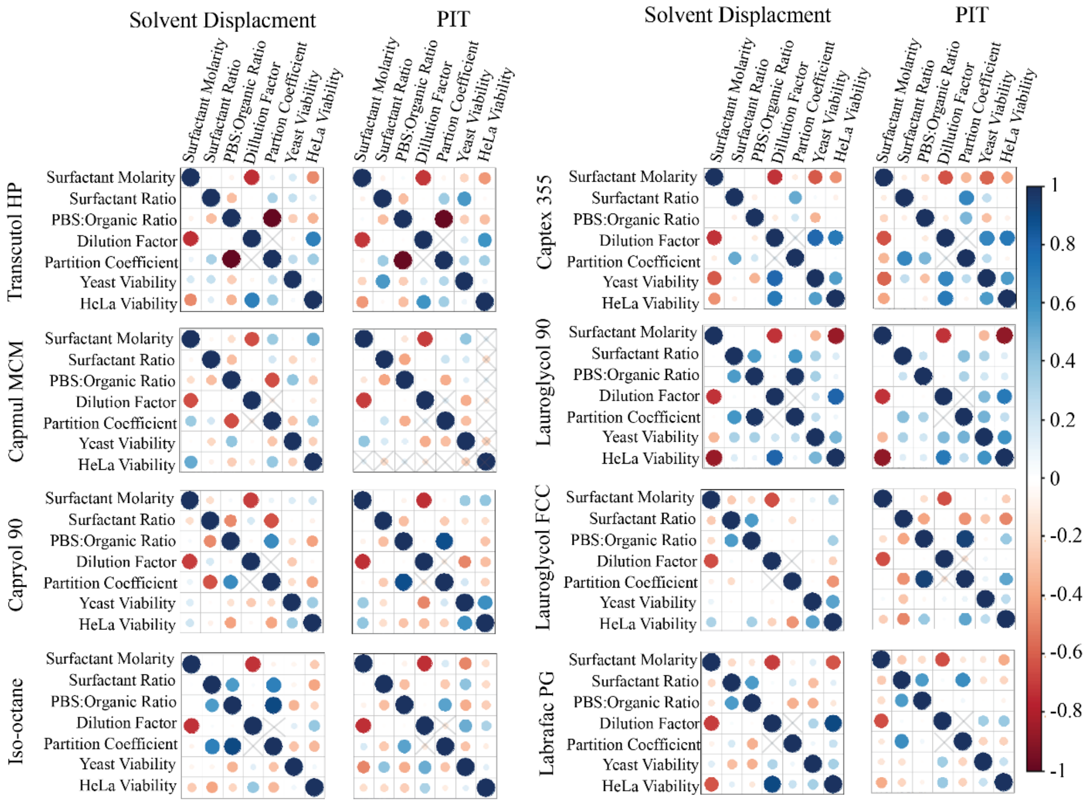

Figure S1: Correlation Analysis by Solvent. The Pearson's correlation is shown for each pairwise set of compositional variables and performance measurements for each solvent and preparation method. Circle size is scaled by strength of the correlation, and the correlation coefficient value is indicated by color code as indicated by the color bar at right. Each grid represents a subset of 324 data points from 36 sample/dilutions. Correlations that were unreliable due to lack of sufficient variance in the data set (e.g. all HeLa cell treatments with Capmul MCM resulted in zero viability) are indicated by boxes with X's.

Table S1: Screening Approach. The structure of the screening approach used for each solvent is presented. 'Optimal' conditions were determined based on maximum encapsulation efficiency from each sample set.

Sample #	Method Used	Surfactant Molarity (mM)	Surfactant Ratio (10MAG:LDAO)	PBS :Organic Ratio
1-4	Solvent Displacement	75,50,35,25	40/60	25/75
5-8	PIT	75,50,35,25	40/60	25/75
9-12	Solvent Displacement	Optimal	60/40, 50/50, 35/65, 30/70	25/75
13-16	PIT	Optimal	60/40, 50/50, 35/65, 30/70	25/75
17-20	Solvent Displacement	Optimal	Optimal	35/65, 50/50, 65/35, 70/30
21-24	PIT	Optimal	Optimal	35/65, 50/50, 65/35, 70/30

Table S2: Formulation Compositions and Measurements.

Solvent	Method	Sample	Surfactant Molarity (mM)	10MAG:LDAO Ratio	PBS:Organic Ratio	Hexanol (uM)	Partition Coefficient	Yeast Viability	HeLa Viability (Fluorescence Emission at 590 nm)
Iso-octane	Solvent Displacement	1	75	40/60	25/75 (vol%)	1062.36	1.31%	0	6.849
		1 (5x)	15	40/60	25/75 (vol%)	212.47	1.31%	0	7.689
		1 (25x)	3	40/60	25/75 (vol%)	42.49	1.31%	25	20.294
		2	50	40/60	25/75 (vol%)	796.77	2.22%	0	4.748
		2 (5x)	10	40/60	25/75 (vol%)	159.35	2.22%	25	6.008
		2 (25x)	2	40/60	25/75 (vol%)	31.87	2.22%	25	6.849
		3	35	40/60	25/75 (vol%)	531.18	1.34%	0	8.109
		3 (5x)	7	40/60	25/75 (vol%)	106.24	1.34%	25	18.613
		3 (25x)	1.4	40/60	25/75 (vol%)	21.25	1.34%	25	100.000
		4	25	40/60	25/75 (vol%)	531.18	1.03%	100	7.269
		4 (5x)	5	40/60	25/75 (vol%)	106.24	1.03%	0	66.513
		4 (25x)	1	40/60	25/75 (vol%)	21.25	1.03%	25	100.000
	PIT	5	75	40/60	25/75 (vol%)	743.65	10.96%	0	9.370
		5 (5x)	15	40/60	25/75 (vol%)	148.73	10.96%	0	8.109
		5 (25x)	3	40/60	25/75 (vol%)	29.75	10.96%	50	8.109
		6	50	40/60	25/75 (vol%)	863.17	4.43%	0	6.430
		6 (5x)	10	40/60	25/75 (vol%)	172.63	4.43%	0	6.008
		6 (25x)	2	40/60	25/75 (vol%)	34.53	4.43%	0	6.849
		7	35	40/60	25/75 (vol%)	743.65	2.23%	0	7.269
		7 (5x)	7	40/60	25/75 (vol%)	148.73	2.23%	0	7.269
		7 (25x)	1.4	40/60	25/75 (vol%)	29.75	2.23%	25	46.765
		8	25	40/60	25/75 (vol%)	531.18	0.56%	0	8.950
		8 (5x)	5	40/60	25/75 (vol%)	106.24	0.56%	25	13.992
		8 (25x)	1	40/60	25/75 (vol%)	21.25	0.56%	0	100.000
	S.D	9	75	60/40	25/75 (vol%)	531.18	3.33%	100	5.168
		9 (5x)	15	60/40	25/75 (vol%)	106.24	3.33%	0	5.588

Iso-octane		9 (25x)	3	60/40	25/75 (vol%)	21.25	3.33%	100	5.168
		10	75	50/50	25/75 (vol%)	1062.36	6.30%	0	4.328
		10 (5x)	15	50/50	25/75 (vol%)	212.47	6.30%	25	4.748
		10 (25x)	3	50/50	25/75 (vol%)	42.49	6.30%	25	4.328
	Solvent Displ.	11	75	35/65	25/75 (vol%)	1062.36	2.63%	0	4.748
		11 (5x)	15	35/65	25/75 (vol%)	212.47	2.63%	25	4.748
		11 (25x)	3	35/65	25/75 (vol%)	42.49	2.63%	50	3.067
		12	75	30/70	25/75 (vol%)	1062.36	0.99%	0	5.168
		12 (5x)	15	30/70	25/75 (vol%)	212.47	0.99%	25	3.908
		12 (25x)	3	30/70	25/75 (vol%)	42.49	0.99%	25	100.000
	PIT	13	50	60/40	25/75 (vol%)	1062.36	0.61%	0	1.387
		13 (5x)	10	60/40	25/75 (vol%)	212.47	0.61%	50	1.807
		13 (25x)	2	60/40	25/75 (vol%)	42.49	0.61%	50	1.387
		14	50	50/50	25/75 (vol%)	876.45	0.80%	0	1.807
		14 (5x)	10	50/50	25/75 (vol%)	175.29	0.80%	25	1.807
		14 (25x)	2	50/50	25/75 (vol%)	35.06	0.80%	50	2.227
		15	50	35/65	25/75 (vol%)	863.17	0.33%	0	2.227
		15 (5x)	10	35/65	25/75 (vol%)	172.63	0.33%	50	2.647
		15 (25x)	2	35/65	25/75 (vol%)	34.53	0.33%	50	2.227
		16	50	30/70	25/75 (vol%)	1195.16	0.57%	0	3.067
		16 (5x)	10	30/70	25/75 (vol%)	239.03	0.57%	50	2.647
		16 (25x)	2	30/70	25/75 (vol%)	47.81	0.57%	25	69.034
	Solvent Displacement	17	75	60/40	35/65 (vol%)	796.77	4.82%	0	1.807
		17 (5x)	15	60/40	35/65 (vol%)	159.35	4.82%	0	1.807
		17 (25x)	3	60/40	35/65 (vol%)	31.87	4.82%	0	1.807
		18	75	60/40	50/50 (vol%)	796.77	5.62%	0	2.227
		18 (5x)	15	60/40	50/50 (vol%)	159.35	5.62%	0	2.227
		18 (25x)	3	60/40	50/50 (vol%)	31.87	5.62%	0	0.966
		19	75	60/40	65/35 (vol%)	796.77	9.87%	0	2.227
		19 (5x)	15	60/40	65/35 (vol%)	159.35	9.87%	0	1.807
		19 (25x)	3	60/40	65/35 (vol%)	31.87	9.87%	0	1.387
		20	75	60/40	70/30 (vol%)	796.77	15.74%	0	2.227
		20 (5x)	15	60/40	70/30 (vol%)	159.35	15.74%	0	2.227

		20 (25x)	3	60/40	70/30 (vol%)	31.87	15.74%	0	32.899
	PIT	21	50	35/65	35/65 (vol%)	929.57	0.67%	0	0.546
		21 (5x)	10	35/65	35/65 (vol%)	185.91	0.67%	0	0.966
		21 (25x)	2	35/65	35/65 (vol%)	37.18	0.67%	0	0.546
		22	50	35/65	50/50 (vol%)	796.77	1.58%	0	0.546
		22 (5x)	10	35/65	50/50 (vol%)	159.35	1.58%	0	0.546
		22 (25x)	2	35/65	50/50 (vol%)	31.87	1.58%	0	0.126
		23	50	35/65	65/35 (vol%)	663.98	8.79%	0	0.966
		23 (5x)	10	35/65	65/35 (vol%)	132.80	8.79%	0	0.546
		23 (25x)	2	35/65	65/35 (vol%)	26.56	8.79%	0	0.126
		24	50	35/65	70/30 (vol%)	584.30	9.65%	0	0.966
		24 (5x)	10	35/65	70/30 (vol%)	116.86	9.65%	0	0.546
		24 (25x)	2	35/65	70/30 (vol%)	23.37	9.65%	0	21.134
**Captex 335**	Solvent Displacement	29	75	40/60	50/50 (vol%)	531.18	5.28%	0	1.072
		29 (5x)	15	40/60	50/50 (vol%)	106.24	5.28%	0	0.919
		29 (25x)	3	40/60	50/50 (vol%)	21.25	5.28%	100	40.123
		30	50	40/60	50/50 (vol%)	1327.95	8.18%	0	1.685
		30 (5x)	10	40/60	50/50 (vol%)	265.59	8.18%	0	1.225
		30 (25x)	2	40/60	50/50 (vol%)	53.12	8.18%	100	29.862
		31	35	40/60	50/50 (vol%)	796.77	21.93%	0	1.072
		31 (5x)	7	40/60	50/50 (vol%)	159.35	21.93%	0	1.378
		31 (25x)	1.4	40/60	50/50 (vol%)	31.87	21.93%	100	100.000
		32	25	40/60	50/50 (vol%)	1327.95	12%	0	0.000
		32 (5x)	5	40/60	50/50 (vol%)	265.59	12%	0	0.000
		32 (25x)	1	40/60	50/50 (vol%)	53.12	12%	100	51.302
	PIT	33	75	40/60	50/50 (vol%)	0.00	23.83%	0	0.153
		33(5x)	15	40/60	50/50 (vol%)	0.00	23.83%	0	0.000
		33 (25x)	3	40/60	50/50 (vol%)	0.00	23.83%	0	0.919
		34	50	40/60	50/50 (vol%)	0.00	12.72%	0	0.459
		34 (5x)	10	40/60	50/50 (vol%)	0.00	12.72%	0	0.766
		34 (25x)	2	40/60	50/50 (vol%)	0.00	12.72%	100	19.602
		35	35	40/60	50/50 (vol%)	0.00	13.84%	0	0.153
		35 (5x)	7	40/60	50/50 (vol%)	0.00	13.84%	0	1.378

Captex 335		35 (25x)	1.4	40/60	50/50 (vol%)	0.00	13.84%	100	100.000
		36	25	40/60	50/50 (vol%)	0.00	6.82%	0	0.000
		36 (5x)	5	40/60	50/50 (vol%)	0.00	6.82%	100	0.000
		36 (25x)	1	40/60	50/50 (vol%)	0.00	6.82%	100	24.962
	Solvent Displacement	37	75	60/40	50/50 (vol%)	398.39	23.48%	0	0.000
		37 (5x)	15	60/40	50/50 (vol%)	79.68	23.48%	100	0.000
		37 (25x)	3	60/40	50/50 (vol%)	15.94	23.48%	100	29.250
		38	75	50/50	50/50 (vol%)	929.57	38.25%	0	0.000
		38 (5x)	15	50/50	50/50 (vol%)	185.91	38.25%	0	0.000
		38 (25x)	3	50/50	50/50 (vol%)	37.18	38.25%	100	2.297
		39	75	35/65	50/50 (vol%)	929.57	16.58%	0	0.000
		39 (5x)	15	35/65	50/50 (vol%)	185.91	16.58%	100	0.000
		39 (25x)	3	35/65	50/50 (vol%)	37.18	16.58%	100	76.723
		40	75	30/70	50/50 (vol%)	929.57	25.92%	0	0.000
		40 (5x)	15	30/70	50/50 (vol%)	185.91	25.92%	100	0.000
		40 (25x)	3	30/70	50/50 (vol%)	37.18	25.92%	100	24.043
	PIT	41	25	60/40	50/50 (vol%)	0.00	44.94%	50	0.000
		41 (5x)	5	60/40	50/50 (vol%)	0.00	44.94%	100	3.369
		41 (25x)	1	60/40	50/50 (vol%)	0.00	44.94%	100	34.916
		42	25	50/50	50/50 (vol%)	0.00	45.29%	0	0.000
		42 (5x)	5	50/50	50/50 (vol%)	0.00	45.29%	100	0.000
		42 (25x)	1	50/50	50/50 (vol%)	0.00	45.29%	100	24.655
		43	25	35/65	50/50 (vol%)	0.00	29.74%	0	0.000
		43 (5x)	5	35/65	50/50 (vol%)	0.00	29.74%	100	0.000
		43 (25x)	1	35/65	50/50 (vol%)	0.00	29.74%	100	60.643
		44	25	30/70	50/50 (vol%)	0.00	25.19%	0	0.000
		44 (5x)	5	30/70	50/50 (vol%)	0.00	25.19%	100	0.000
		44 (25x)	1	30/70	50/50 (vol%)	0.00	25.19%	100	5.513
	Solvent	45	75	30/70	25/75 (vol%)	1063.36	2.80%	50	0.000
		45 (5x)	15	30/70	25/75 (vol%)	212.67	2.80%	100	0.000
		45 (25x)	3	30/70	25/75 (vol%)	42.53	2.80%	100	24.809
		46	75	30/70	35/65 (vol%)	929.57	2.40%	0	0.000

**Captex 335**		46 (5x)	15	30/70	35/65 (vol%)	185.91	2.40%	0	0.000
		46 (25x)	3	30/70	35/65 (vol%)	37.18	2.40%	100	10.413
		47	75	30/70	65/35 (vol%)	796.77	9.65%	0	0.000
		47 (5x)	15	30/70	65/35 (vol%)	159.35	9.65%	0	0.000
		47 (25x)	3	30/70	65/35 (vol%)	31.87	9.65%	100	54.977
		48	75	30/70	70/30 (vol%)	796.77	20.28%	0	0.000
		48 (5x)	15	30/70	70/30 (vol%)	159.35	20.28%	0	0.000
		48 (25x)	3	30/70	70/30 (vol%)	31.87	20.28%	0	0.000
	PIT	49	75	30/70	25/75 (vol%)	0.00	6.05%	0	0.000
		49 (5x)	15	30/70	25/75 (vol%)	0.00	6.05%	100	1.378
		49 (25x)	3	30/70	25/75 (vol%)	0.00	6.05%	100	42.879
		50	25	30/70	35/65 (vol%)	0.00	8.01%	0	0.000
		50 (5x)	5	30/70	35/65 (vol%)	0.00	8.01%	0	3.828
		50 (25x)	1	30/70	35/65 (vol%)	0.00	8.01%	100	23.890
		51	25	30/70	65/35 (vol%)	0.00	29.89%	0	0.000
		51 (5x)	5	30/70	65/35 (vol%)	0.00	29.89%	0	0.000
		51 (25x)	1	30/70	65/35 (vol%)	0.00	29.89%	100	41.960
		52	25	30/70	70/30 (vol%)	0.00	34.24%	0	0.000
		52 (5x)	5	30/70	70/30 (vol%)	0.00	34.24%	0	0.000
		52 (25x)	1	30/70	70/30 (vol%)	0.00	34.24%	50	8.423
**Capmul MCM**	Solvent Displ.	57	75	40/60	25/75 (vol%)	1195.16	83.73%	0	0.752
		57(5x)	15	40/60	25/75 (vol%)	239.03	83.73%	0	0.752
		57(25x)	3	40/60	25/75 (vol%)	47.81	83.73%	0	0.752
		58	50	40/60	25/75 (vol%)	1991.93	78.73%	0	0.451
		58(5x)	10	40/60	25/75 (vol%)	398.39	78.73%	0	0.150
		58(25x)	2	40/60	25/75 (vol%)	79.68	78.73%	0	0.451
	Solvent Displ.	59	35	40/60	25/75 (vol%)	1195.16	89.14%	0	0.150
		59(5x)	7	40/60	25/75 (vol%)	239.03	89.14%	0	0.150
		59(25x)	1.4	40/60	25/75 (vol%)	47.81	89.14%	0	0.000
		60	25	40/60	25/75 (vol%)	1593.54	85.98%	0	0.000
		60(5x)	5	40/60	25/75 (vol%)	318.71	85.98%	0	0.000
		60(25x)	1	40/60	25/75 (vol%)	63.74	85.98%	0	0.000
**Ca**	P	61	75	40/60	25/75 (vol%)	0.00	77.47%	0	0.000

		61(5x)	15	40/60	25/75 (vol%)	0.00	77.47%	0	0.000
		61(25x)	3	40/60	25/75 (vol%)	0.00	77.47%	0	0.000
		62	50	40/60	25/75 (vol%)	0.00	86.90%	0	0.000
		62(5x)	10	40/60	25/75 (vol%)	0.00	86.90%	0	0.000
		62(25x)	2	40/60	25/75 (vol%)	0.00	86.90%	0	0.000
		63	35	40/60	25/75 (vol%)	0.00	82.27%	0	0.000
		63(5x)	7	40/60	25/75 (vol%)	0.00	82.27%	0	0.000
		63(25x)	1.4	40/60	25/75 (vol%)	0.00	82.27%	0	0.000
		64	25	40/60	25/75 (vol%)	0.00	51.78%	0	0.000
		64(5x)	5	40/60	25/75 (vol%)	0.00	51.78%	0	0.000
		64(25x)	1	40/60	25/75 (vol%)	0.00	51.78%	0	0.000
	Solvent Displacement	65	25	60/40	25/75 (vol%)	1195.16	80.52%	0	0.000
		65(5x)	5	60/40	25/75 (vol%)	239.03	80.52%	0	0.000
		65(25x)	1	60/40	25/75 (vol%)	47.81	80.52%	0	0.000
		66	25	50/50	25/75 (vol%)	1991.93	63.36%	0	0.000
		66(5x)	5	50/50	25/75 (vol%)	398.39	63.36%	25	0.000
		66(25x)	1	50/50	25/75 (vol%)	79.68	63.36%	0	0.000
		67	25	35/65	25/75 (vol%)	1991.93	63.12%	0	0.000
		67(5x)	5	35/65	25/75 (vol%)	398.39	63.12%	0	0.000
		67(25x)	1	35/65	25/75 (vol%)	79.68	63.12%	0	0.000
		68	25	30/70	25/75 (vol%)	1991.93	76.75%	0	0.000
		68(5x)	5	30/70	25/75 (vol%)	398.39	76.75%	0	0.000
		68(25x)	1	30/70	25/75 (vol%)	79.68	76.75%	25	0.000
	PIT	69	35	60/40	25/75 (vol%)	0.00	66.86%	100	0.000
		69(5x)	7	60/40	25/75 (vol%)	0.00	66.86%	0	0.000
		69(25x)	1.4	60/40	25/75 (vol%)	0.00	66.86%	25	0.000
		70	35	50/50	25/75 (vol%)	0.00	89.85%	50	0.000
		70(5x)	7	50/50	25/75 (vol%)	0.00	89.85%	0	0.000
		70(25x)	1.4	50/50	25/75 (vol%)	0.00	89.85%	0	0.000
		71	35	35/65	25/75 (vol%)	0.00	100%	50	0.000
		71(5x)	7	35/65	25/75 (vol%)	0.00	100%	0	0.000
**Capmu**		71(25x)	1.4	35/65	25/75 (vol%)	0.00	100%	0	0.000

		72	35	30/70	25/75 (vol%)	0.00	81.29%	50	0.000
		72(5x)	7	30/70	25/75 (vol%)	0.00	81.29%	0	0.000
		72(25x)	1.4	30/70	25/75 (vol%)	0.00	81.29%	0	0.000
	Solvent Displacement	73	25	35/65	35/65 (vol%)	796.77	79.23%	25	0.000
		73(5x)	5	35/65	35/65 (vol%)	159.35	79.23%	0	0.000
		73(25x)	1	35/65	35/65 (vol%)	31.87	79.23%	0	0.000
		74	25	35/65	50/50 (vol%)	796.77	69.92%	25	0.000
		74(5x)	5	35/65	50/50 (vol%)	159.35	69.92%	0	0.000
		74(25x)	1	35/65	50/50 (vol%)	31.87	69.92%	0	0.000
		75	25	35/65	65/35 (vol%)	796.77	65.59%	50	0.000
		75(5x)	5	35/65	65/35 (vol%)	159.35	65.59%	0	0.000
		75(25x)	1	35/65	65/35 (vol%)	31.87	65.59%	25	0.000
		76	25	35/65	70/30 (vol%)	796.77	49.20%	0	0.000
		76(5x)	5	35/65	70/30 (vol%)	159.35	49.20%	0	0.000
		76(25x)	1	35/65	70/30 (vol%)	31.87	49.20%	25	0.000
	PIT	77	35	30/70	35/65 (vol%)	0.00	22.81%	75	0.000
		77(5x)	7	30/70	35/65 (vol%)	0.00	22.81%	0	0.000
		77(25x)	1.4	30/70	35/65 (vol%)	0.00	22.81%	0	0.000
		78	35	30/70	50/50 (vol%)	0.00	79.32%	100	0.000
		78(5x)	7	30/70	50/50 (vol%)	0.00	79.32%	0	0.000
		78(25x)	1.4	30/70	50/50 (vol%)	0.00	79.32%	0	0.000
		79	35	30/70	65/35 (vol%)	0.00	46.14%	100	0.000
		79(5x)	7	30/70	65/35 (vol%)	0.00	46.14%	0	0.000
		79(25x)	1.4	30/70	65/35 (vol%)	0.00	46.14%	0	0.000
		80	35	30/70	70/30 (vol%)	0.00	69.73%	50	0.000
		80(5x)	7	30/70	70/30 (vol%)	0.00	69.73%	0	0.000
		80(25x)	1.4	30/70	70/30 (vol%)	0.00	69.73%	0	0.000
**Lauroglycol 90**	Solvent	85	75	40/60	25/75 (vol%)	2390.31	7.39%	0	0.724
		85 (5x)	15	40/60	25/75 (vol%)	478.06	7.39%	0	25.129
		85 (25x)	3	40/60	25/75 (vol%)	95.61	7.39%	0	100.000
		86	50	40/60	25/75 (vol%)	2390.31	5.97%	0	0.620
		86 (5x)	10	40/60	25/75 (vol%)	478.06	5.97%	100	100.000
		86 (25x)	2	40/60	25/75 (vol%)	95.61	5.97%	0	83.454

		87	35	40/60	25/75 (vol%)	2390.31	7.73%	0	0.103
		87 (5x)	7	40/60	25/75 (vol%)	478.06	7.73%	0	56.774
		87 (25x)	1.4	40/60	25/75 (vol%)	95.61	7.73%	0	100.000
		88	25	40/60	25/75 (vol%)	2390.31	7.08%	0	0.000
		88 (5x)	5	40/60	25/75 (vol%)	478.06	7.08%	100	50.569
		88 (25x)	1	40/60	25/75 (vol%)	95.61	7.08%	100	79.938
	PIT	89	75	40/60	25/75 (vol%)	0.00	8.14%	0	0.000
		89 (5x)	15	40/60	25/75 (vol%)	0.00	8.14%	100	70.321
		89 (25x)	3	40/60	25/75 (vol%)	0.00	8.14%	100	100.000
		90	50	40/60	25/75 (vol%)	0.00	7.55%	0	0.207
		90 (5x)	10	40/60	25/75 (vol%)	0.00	7.55%	0	65.460
		90 (25x)	2	40/60	25/75 (vol%)	0.00	7.55%	0	100.000
		91	35	40/60	25/75 (vol%)	0.00	8.29%	0	0.310
		91 (5x)	7	40/60	25/75 (vol%)	0.00	8.29%	50	82.834
		91 (25x)	1.4	40/60	25/75 (vol%)	0.00	8.29%	100	100.000
		92	25	40/60	25/75 (vol%)	0.00	7.47%	50	0.000
		92 (5x)	5	40/60	25/75 (vol%)	0.00	7.47%	0	100.000
		92 (25x)	1	40/60	25/75 (vol%)	0.00	7.47%	0	100.000
	S.D.	93	75	60/40	25/75 (vol%)	2655.90	6.35%	0	0.000
		93 (5x)	15	60/40	25/75 (vol%)	531.18	6.35%	100	90.693
		93 (25x)	3	60/40	25/75 (vol%)	106.24	6.35%	0	100.000
		94	75	50/50	25/75 (vol%)	2655.90	5.95%	100	0.000
		94 (5x)	15	50/50	25/75 (vol%)	531.18	5.95%	100	65.460
		94 (25x)	3	50/50	25/75 (vol%)	106.24	5.95%	100	85.109
	Solvent Displ.	95	75	35/65	25/75 (vol%)	2655.90	6.47%	100	0.000
		95 (5x)	15	35/65	25/75 (vol%)	531.18	6.47%	100	52.844
		95 (25x)	3	35/65	25/75 (vol%)	106.24	6.47%	100	100.000
		96	75	30/70	25/75 (vol%)	2655.90	4.72%	50	0.000
		96 (5x)	15	30/70	25/75 (vol%)	531.18	4.72%	100	32.989
		96 (25x)	3	30/70	25/75 (vol%)	106.24	4.72%	100	79.835
**Laugrolyc**	PIT	97	75	60/40	25/75 (vol%)	0.00	16.73%	100	4.100
		97 (5x)	15	60/40	25/75 (vol%)	0.00	16.73%	100	90.196
		97 (25x)	3	60/40	25/75 (vol%)	0.00	16.73%	100	100.000

	98	75	50/50	25/75 (vol%)	0.00	21.78%	100	3.209
	98 (5x)	15	50/50	25/75 (vol%)	0.00	21.78%	100	100.000
	98 (25x)	3	50/50	25/75 (vol%)	0.00	21.78%	100	100.000
	99	75	35/65	25/75 (vol%)	0.00	18.35%	100	5.526
	99 (5x)	15	35/65	25/75 (vol%)	0.00	18.35%	100	100.000
	99 (25x)	3	35/65	25/75 (vol%)	0.00	18.35%	100	100.000
	100	75	30/70	25/75 (vol%)	0.00	11.03%	100	5.704
	100 (5x)	15	30/70	25/75 (vol%)	0.00	11.03%	100	49.911
	100 (25x)	3	30/70	25/75 (vol%)	0.00	11.03%	100	100.000
Solvent Displacement	101	75	60/40	35/65 (vol%)	1062.36	8.26%	100	0.000
	101 (5x)	15	60/40	35/65 (vol%)	212.47	8.26%	100	59.979
	101 (25x)	3	60/40	35/65 (vol%)	42.49	8.26%	100	91.003
	102	75	60/40	50/50 (vol%)	1062.36	11.01%	50	0.000
	102 (5x)	15	60/40	50/50 (vol%)	212.47	11.01%	100	97.725
	102 (25x)	3	60/40	50/50 (vol%)	42.49	11.01%	100	89.038
	103	75	60/40	65/35 (vol%)	1062.36	16.02%	50	0.000
	103 (5x)	15	60/40	65/35 (vol%)	212.47	16.02%	100	72.802
	103 (25x)	3	60/40	65/35 (vol%)	42.49	16.02%	100	99.586
	104	75	60/40	70/30 (vol%)	1062.36	18.59%	0	0.000
	104 (5x)	15	60/40	70/30 (vol%)	212.47	18.59%	100	65.977
	104 (25x)	3	60/40	70/30 (vol%)	42.49	18.59%	100	72.389
PIT	105	75	50/50	35/65 (vol%)	0.00	37.07%	100	5.348
	105 (5x)	15	50/50	35/65 (vol%)	0.00	37.07%	100	100.000
	105 (25x)	3	50/50	35/65 (vol%)	0.00	37.07%	100	100.000
	106	75	50/50	50/50 (vol%)	0.00	10.10%	100	4.278
PIT	106 (5x)	15	50/50	50/50 (vol%)	0.00	10.10%	100	100.000
	106 (25x)	3	50/50	50/50 (vol%)	0.00	10.10%	100	100.000
	107	75	50/50	65/35 (vol%)	0.00	21.92%	75	5.348
	107 (5x)	15	50/50	65/35 (vol%)	0.00	21.92%	100	100.000
	107 (25x)	3	50/50	65/35 (vol%)	0.00	21.92%	100	100.000
	108	75	50/50	70/30 (vol%)	0.00	24.73%	100	5.169
	108 (5x)	15	50/50	70/30 (vol%)	0.00	24.73%	100	96.257
	108 (25x)	3	50/50	70/30 (vol%)	0.00	24.73%	100	100.000

Lauroglycol FCC	Solvent Displacement	169	75	40/60	25/75 (vol%)	132.80	9.91%	100	100.000
		169 (5x)	15	40/60	25/75 (vol%)	26.56	9.91%	100	100.000
		169 (25x)	3	40/60	25/75 (vol%)	5.31	9.91%	100	100.000
		170	50	40/60	25/75 (vol%)	132.80	6.11%	100	100.000
		170 (5x)	10	40/60	25/75 (vol%)	26.56	6.11%	100	100.000
		170 (25x)	2	40/60	25/75 (vol%)	5.31	6.11%	100	100.000
		171	35	40/60	25/75 (vol%)	132.80	6.48%	100	100.000
		171 (5x)	7	40/60	25/75 (vol%)	26.56	6.48%	100	100.000
		171 (25x)	1.4	40/60	25/75 (vol%)	5.31	6.48%	100	100.000
		172	25	40/60	25/75 (vol%)	132.80	3.65%	100	100.000
		172 (5x)	5	40/60	25/75 (vol%)	26.56	3.65%	100	100.000
		172 (25x)	1	40/60	25/75 (vol%)	5.31	3.65%	100	100.000
	PIT	173	75	40/60	25/75 (vol%)	0.00	2.67%	100	10.745
		173 (5x)	15	40/60	25/75 (vol%)	0.00	2.67%	100	100.000
		173 (25x)	3	40/60	25/75 (vol%)	0.00	2.67%	100	100.000
		174	50	40/60	25/75 (vol%)	0.00	3.63%	100	100.000
		174 (5x)	10	40/60	25/75 (vol%)	0.00	3.63%	100	100.000
		174 (25x)	2	40/60	25/75 (vol%)	0.00	3.63%	100	61.461
		175	35	40/60	25/75 (vol%)	0.00	3.53%	100	100.000
		175 (5x)	7	40/60	25/75 (vol%)	0.00	3.53%	100	100.000
		175 (25x)	1.4	40/60	25/75 (vol%)	0.00	3.53%	100	100.000
		176	25	40/60	25/75 (vol%)	0.00	3.79%	100	100.000
		176 (5x)	5	40/60	25/75 (vol%)	0.00	3.79%	100	100.000
		176(25x)	1	40/60	25/75 (vol%)	0.00	3.79%	100	100.000
	Solvent Displacement	177	25	60/40	25/75 (vol%)	132.80	12.97%	50	69.074
		177 (5x)	5	60/40	25/75 (vol%)	26.56	12.97%	100	68.172
		177 (25x)	1	60/40	25/75 (vol%)	5.31	12.97%	100	30.926
		178	25	50/50	25/75 (vol%)	132.80	7.30%	100	65.237
		178 (5x)	5	50/50	25/75 (vol%)	26.56	7.30%	25	32.506
		178 (25x)	1	50/50	25/75 (vol%)	5.31	7.30%	25	39.729
		179	25	35/65	25/75 (vol%)	132.80	16.24%	25	67.269
		179 (5x)	5	35/65	25/75 (vol%)	26.56	16.24%	100	57.788
		179 (25x)	1	35/65	25/75 (vol%)	5.31	16.24%	100	80.135

Lauroglycol FCC		180	25	30/70	25/75 (vol%)	132.80	7.99%	100	86.230
		180 (5x)	5	30/70	25/75 (vol%)	26.56	7.99%	25	55.530
		180 (25x)	1	30/70	25/75 (vol%)	5.31	7.99%	25	63.431
	PIT	181	25	60/40	25/75 (vol%)	0.00	1.37%	25	60.722
		181 (5x)	5	60/40	25/75 (vol%)	0.00	1.37%	100	46.050
		181 (25x)	1	60/40	25/75 (vol%)	0.00	1.37%	100	39.052
		182	25	50/50	25/75 (vol%)	0.00	1.00%	100	58.465
		182 (5x)	5	50/50	25/75 (vol%)	0.00	1.00%	0	45.824
		182 (25x)	1	50/50	25/75 (vol%)	0.00	1.00%	25	69.752
		183	25	35/65	25/75 (vol%)	0.00	1.63%	0	61.625
		183 (5x)	5	35/65	25/75 (vol%)	0.00	1.63%	100	71.783
		183 (25x)	1	35/65	25/75 (vol%)	0.00	1.63%	100	83.973
		184	25	30/70	25/75 (vol%)	0.00	1.40%	100	46.050
		184 (5x)	5	30/70	25/75 (vol%)	0.00	1.40%	0	45.824
		184 (25x)	1	30/70	25/75 (vol%)	0.00	1.40%	50	33.183
	Solvent Dis.	185	25	60/40	35/65 (vol%)	0.00	2.64	100	100.000
		185 (5x)	5	60/40	35/65 (vol%)	0.00	2.64	100	100.000
		185 (25x)	1	60/40	35/65 (vol%)	0.00	2.64	100	100.000
		186	25	60/40	50/50 (vol%)	0.00	2.34	50	100.000
		186 (5x)	5	60/40	50/50 (vol%)	0.00	2.34	100	100.000
		186 (25x)	1	60/40	50/50 (vol%)	0.00	2.34	100	100.000
	Solvent Displ.	187	25	60/40	65/35 (vol%)	0.00	8.1	25	100.000
		187 (5x)	5	60/40	65/35 (vol%)	0.00	8.1	100	100.000
		187 (25x)	1	60/40	65/35 (vol%)	0.00	8.1	100	100.000
		188	25	60/40	70/30 (vol%)	0.00	11.24	50	100.000
		188 (5x)	5	60/40	70/30 (vol%)	0.00	11.24	100	100.000
		188 (25x)	1	60/40	70/30 (vol%)	0.00	11.24	100	100.000
	PIT	189	25	30/70	35/65 (vol%)	0.00	2.72	100	100.000
		189 (5x)	5	30/70	35/65 (vol%)	0.00	2.72	100	100.000
		189 (25x)	1	30/70	35/65 (vol%)	0.00	2.72	100	100.000
		190	25	30/70	50/50 (vol%)	0.00	3.34	50	79.073
		190 (5x)	5	30/70	50/50 (vol%)	0.00	3.34	100	96.629
		190 (25x)	1	30/70	50/50 (vol%)	0.00	3.34	100	100.000

		191	25	30/70	65/35 (vol%)	0.00	8.01	0	100.000
		191 (5x)	5	30/70	65/35 (vol%)	0.00	8.01	100	100.000
		191 (25x)	1	30/70	65/35 (vol%)	0.00	8.01	100	100.000
		192	25	30/70	70/30 (vol%)	0.00	10.5	50	100.000
		192 (5x)	5	30/70	70/30 (vol%)	0.00	10.5	100	100.000
		192 (25x)	1	30/70	70/30 (vol%)	0.00	10.5	100	100.000
**Capryol 90**	Solvent Displacement	197	75	40/60	25/75 (vol%)	1327.95	13.35%	0	6.732
		197 (5x)	15	40/60	25/75 (vol%)	331.99	13.35%	0	7.061
		197 (25x)	3	40/60	25/75 (vol%)	53.12	13.35%	0	6.404
		198	50	40/60	25/75 (vol%)	132.80	8.44%	0	7.718
		198 (25x)	10	40/60	25/75 (vol%)	26.56	8.44%	0	5.747
		198 (5x)	2	40/60	25/75 (vol%)	1.06	8.44%	0	6.240
		199	35	40/60	25/75 (vol%)	132.80	10.02%	100	6.076
		199 (5x)	7	40/60	25/75 (vol%)	26.56	10.02%	100	5.090
		199 (25x)	1.4	40/60	25/75 (vol%)	1.06	10.02%	0	5.747
		200	25	40/60	25/75 (vol%)	132.80	8.74%	50	6.404
		200 (5x)	5	40/60	25/75 (vol%)	26.56	8.74%	0	4.598
		200 (25x)	1	40/60	25/75 (vol%)	1.06	8.74%	0	6.240
	PIT	201	75	40/60	25/75 (vol%)	0.00	11.89%	50	10.016
		201 (5x)	15	40/60	25/75 (vol%)	0.00	11.89%	0	9.031
		201 (25x)	3	40/60	25/75 (vol%)	0.00	11.89%	0	6.240
		202	50	40/60	25/75 (vol%)	0.00	11.95%	50	8.374
		202 (5x)	10	40/60	25/75 (vol%)	0.00	11.95%	50	6.897
		202 (25x)	2	40/60	25/75 (vol%)	0.00	11.95%	0	4.598
		203	35	40/60	25/75 (vol%)	0.00	11.93%	50	6.076
		203 (5x)	7	40/60	25/75 (vol%)	0.00	11.93%	25	7.718
		203 (25x)	1.4	40/60	25/75 (vol%)	0.00	11.93%	0	5.255
		204	25	40/60	25/75 (vol%)	0.00	12.85%	50	6.240
		204 (5x)	5	40/60	25/75 (vol%)	0.00	12.85%	50	6.732
		204 (25x)	1	40/60	25/75 (vol%)	0.00	12.85%	0	5.090
	Solvent [illegible]	206	50	50/50	25/75 (vol%)	132.80	8.44%	0	0.573
		206 (5x)	10	50/50	25/75 (vol%)	26.56	8.44%	0	0.287
		206 (25x)	2	50/50	25/75 (vol%)	1.06	8.44%	0	0.430

		207	50	35/65	25/75 (vol%)	132.80	7.20%	0	1.003
		207 (5x)	10	35/65	25/75 (vol%)	26.56	7.20%	0	0.430
		207 (25x)	2	35/65	25/75 (vol%)	1.06	7.20%	0	0.287
		208	50	30/70	25/75 (vol%)	132.80	7.40%	0	0.860
		208 (5x)	10	30/70	25/75 (vol%)	26.56	7.40%	0	0.000
		208 (25x)	2	30/70	25/75 (vol%)	1.06	7.40%	0	0.143
	PIT	209	50	60/40	25/75 (vol%)	0.00	12.40%	0	0.573
		209 (5x)	10	60/40	25/75 (vol%)	0.00	12.40%	0	0.287
		209 (25x)	2	60/40	25/75 (vol%)	0.00	12.40%	0	0.287
		210	50	50/50	25/75 (vol%)	0.00	10.57%	0	0.287
		210 (5x)	10	50/50	25/75 (vol%)	0.00	10.57%	0	0.430
		210 (25x)	2	50/50	25/75 (vol%)	0.00	10.57%	0	0.287
		211	50	35/65	25/75 (vol%)	0.00	10.81%	0	1.289
		211 (5x)	10	35/65	25/75 (vol%)	0.00	10.81%	0	0.143
		211 (5x)	2	35/65	25/75 (vol%)	0.00	10.81%	0	0.287
		212	50	30/70	25/75 (vol%)	0.00	10.94%	0	1.146
		212 (5x)	10	30/70	25/75 (vol%)	0.00	10.94%	0	0.860
		212 (25x)	2	30/70	25/75 (vol%)	0.00	10.94%	0	0.716
**Capryol 90**	Solvent Displacement	213	50	30/70	35/65 (vol%)	132.80	26.17%	0	0.810
		213 (5x)	10	30/70	35/65 (vol%)	26.56	26.17%	0	0.675
		213 (25x)	2	30/70	35/65 (vol%)	1.06	26.17%	0	0.270
		214	50	30/70	50/50 (vol%)	132.80	16.14%	0	0.945
		214 (5x)	10	30/70	50/50 (vol%)	26.56	16.14%	0	0.135
		214 (25x)	2	30/70	50/50 (vol%)	1.06	16.14%	0	0.203
		215	50	30/70	65/35 (vol%)	132.80	22.75%	0	0.878
		215 (5x)	10	30/70	65/35 (vol%)	26.56	22.75%	0	0.743
		215 (25x)	2	30/70	65/35 (vol%)	1.06	22.75%	0	0.405
**Capryol 90**		216	50	30/70	70/30 (vol%)	132.80	31.92%	0	0.878
		216 (5x)	10	30/70	70/30(vol%)	26.56	31.92%	0	0.405
		216 (25x)	2	30/70	70/30 (vol%)	1.06	31.92%	0	0.135
	P	217	50	35/65	35/65 (vol%)	0.00	15.43%	0	1.283

		217 (5x)	10	35/65	35/65 (vol%)	0.00	15.43%	25	1.080
		217 (25x)	2	35/65	35/65 (vol%)	0.00	15.43%	0	0.810
		218	50	35/65	50/50 (vol%)	0.00	14.18%	0	1.621
		218 (5x)	10	35/65	50/50 (vol%)	0.00	14.18%	0	1.283
		218 (25x)	2	35/65	50/50 (vol%)	0.00	14.18%	0	0.810
		219	50	35/65	65/35 (vol%)	0.00	17.11%	0	1.621
		219 (5x)	10	35/65	65/35 (vol%)	0.00	17.11%	0	1.283
		219 (25x)	2	35/65	65/35 (vol%)	0.00	17.11%	0	0.338
		220	50	35/65	70/30 (vol%)	0.00	25.05%	0	0.473
		220 (5x)	10	35/65	70/30(vol%)	0.00	25.05%	50	1.148
		220 (25x)	2	35/65	70/30 (vol%)	0.00	25.05%	0	1.485
**Labrafac PG**	Solvent Dis	225	75	40/60	25/75 (vol%)	1859.13	8.17%	100	4.598
		225 (5x)	15	40/60	25/75 (vol%)	371.83	8.17%	0	6.897
		225 (25x)	3	40/60	25/75 (vol%)	74.37	8.17%	0	50.739
		226	50	40/60	25/75 (vol%)	1859.13	5.08%	100	5.090
		226 (5x)	10	40/60	25/75 (vol%)	371.83	5.08%	0	24.138
		226 (25x)	2	40/60	25/75 (vol%)	74.37	5.08%	0	55.501
	Solvent Displ.	227	35	40/60	25/75 (vol%)	929.57	6.72%	100	5.090
		227 (5x)	7	40/60	25/75 (vol%)	185.91	6.72%	0	66.010
		227 (25x)	1.4	40/60	25/75 (vol%)	37.18	6.72%	100	86.043
		228	25	40/60	25/75 (vol%)	796.77	6.69%	0	4.598
		228 (25x)	5	40/60	25/75 (vol%)	159.35	6.69%	0	76.190
		228 (25x)	1	40/60	25/75 (vol%)	31.87	6.69%	100	64.696
**Labrafac PG**	PIT	229	75	40/60	25/75 (vol%)	0.00	2.02%	50	96.842
		229 (5x)	15	40/60	25/75 (vol%)	0.00	2.02%	100	100.000
		229 (25x)	3	40/60	25/75 (vol%)	0.00	2.02%	100	100.000
		230	50	40/60	25/75 (vol%)	0.00	1.33%	100	100.000
		230 (5x)	10	40/60	25/75 (vol%)	0.00	1.33%	100	100.000
		230 (25x)	2	40/60	25/75 (vol%)	0.00	1.33%	100	99.883
		231	35	40/60	25/75 (vol%)	0.00	1.02%	100	100.000
		231 (5x)	7	40/60	25/75 (vol%)	0.00	1.02%	100	100.000
		231 (25x)	1.4	40/60	25/75 (vol%)	0.00	1.02%	100	100.000
		232	25	40/60	25/75 (vol%)	0.00	0.72%	50	100.000

	232 (5x)	5	40/60	25/75 (vol%)	0.00	0.72%	100	100.000
	232 (25x)	1	40/60	25/75 (vol%)	0.00	0.72%	100	100.000
Solvent Displacement	233	35	60/40	25/75 (vol%)	796.77	50.67%	0	2.456
	233 (5x)	7	60/40	25/75 (vol%)	159.35	50.67%	25	82.105
	233 (25x)	1.4	60/40	25/75 (vol%)	31.87	50.67%	100	100.000
	234	35	50/50	25/75 (vol%)	796.77	6.44%	0	1.404
	234 (5x)	7	50/50	25/75 (vol%)	159.35	6.44%	25	34.620
	234 (25x)	1.4	50/50	25/75 (vol%)	31.87	6.44%	100	89.825
	235	35	35/65	25/75 (vol%)	995.96	22.06%	0	1.520
	235 (5x)	7	35/65	25/75 (vol%)	199.19	22.06%	25	7.368
	235 (25x)	1.4	35/65	25/75 (vol%)	39.84	22.06%	100	100.000
	236	35	30/70	25/75 (vol%)	929.57	24.01%	0	1.287
	236 (5x)	7	30/70	25/75 (vol%)	185.91	24.01%	25	20.468
	236 (25x)	1.4	30/70	25/75 (vol%)	37.18	24.01%	100	87.135
PIT	237	25	60/40	25/75 (vol%)	0.00	25.46%	0	11.902
	237 (5x)	5	60/40	25/75 (vol%)	0.00	25.46%	25	131.775
	237 (25x)	1	60/40	25/75 (vol%)	0.00	25.46%	100	109.883
	238	25	50/50	25/75 (vol%)	0.00	2.23%	0	33.900
	238 (5x)	5	50/50	25/75 (vol%)	0.00	2.23%	50	130.925
	238 (25x)	1	50/50	25/75 (vol%)	0.00	2.23%	100	133.900
	239	25	35/65	25/75 (vol%)	0.00	0.91%	25	41.552
	239 (5x)	5	35/65	25/75 (vol%)	0.00	0.91%	50	122.635
	239 (25x)	1	35/65	25/75 (vol%)	0.00	0.91%	100	111.371
	240	25	30/70	25/75 (vol%)	0.00	0.97%	0	78.108
	240 (5x)	5	30/70	25/75 (vol%)	0.00	0.97%	0	1.148
	240 (25x)	1	30/70	25/75 (vol%)	0.00	0.97%	50	1.485
Solvent	241	35	60/40	35/65 (vol%)	663.98	11.14%	25	11.902
	241 (5x)	7	60/40	35/65 (vol%)	132.80	11.14%	25	131.775
	241 (25x)	1.4	60/40	35/65 (vol%)	26.56	11.14%	50	109.883
	242	35	60/40	50/50 (vol%)	597.58	8.88%	0	33.900
	242 (5x)	7	60/40	50/50 (vol%)	119.52	8.88%	50	130.925
	242 (25x)	1.4	60/40	50/50 (vol%)	23.90	8.88%	0	133.900

**Labrafac PG**		243	35	60/40	65/35 (vol%)	531.18	3.05%	25	41.552
		243 (5x)	7	60/40	65/35 (vol%)	106.24	3.05%	25	122.635
		243 (25x)	1.4	60/40	65/35 (vol%)	21.25	3.05%	0	111.371
		244	35	60/40	70/30 (vol%)	531.18	5.53%	0	78.108
		244 (5x)	7	60/40	70/30(vol%)	106.24	5.53%	0	111.052
		244 (25x)	1.4	60/40	70/30 (vol%)	21.25	5.53%	0	110.840
	PIT	245	25	60/40	35/65 (vol%)	0.00	4.12%	50	0.638
		245 (5x)	5	60/40	35/65 (vol%)	0.00	4.12%	100	1.169
		245 (25x)	1	60/40	35/65 (vol%)	0.00	4.12%	0	116.578
		246	25	60/40	50/50 (vol%)	0.00	5.80%	100	0.744
		246 (5x)	5	60/40	50/50 (vol%)	0.00	5.80%	100	0.744
		246 (25x)	1	60/40	50/50 (vol%)	0.00	5.80%	100	106.057
		247	25	60/40	65/35 (vol%)	0.00	9.60%	75	0.744
		247 (5x)	5	60/40	65/35 (vol%)	0.00	9.60%	75	0.850
		247 (25x)	1	60/40	65/35 (vol%)	0.00	9.60%	100	89.692
		248	25	60/40	70/30 (vol%)	0.00	1.66%	100	0.531
		248 (5x)	5	60/40	70/30(vol%)	0.00	1.66%	25	0.000
		248 (25x)	1	60/40	70/30 (vol%)	0.00	1.66%	0	110.308
**Transcutol HP**	Solvent Displacement	281	75	40/60	25/75 (vol%)	531.18	78.24%	0	4.762
		281 (5x)	15	40/60	25/75 (vol%)	106.24	78.24%	0	9.852
		281 (25x)	3	40/60	25/75 (vol%)	21.25	78.24%	0	44.499
		282	50	40/60	25/75 (vol%)	531.18	79.00%	0	6.732
		282 (5x)	10	40/60	25/75 (vol%)	106.24	79.00%	0	12.644
		282 (25X)	2	40/60	25/75 (vol%)	21.25	79.00%	0	73.892
		283	35	40/60	25/75 (vol%)	531.18	76.61%	0	3.612
		283 (5X)	7	40/60	25/75 (vol%)	106.24	76.61%	0	43.678
		283 (25X)	1.4	40/60	25/75 (vol%)	21.25	76.61%	0	51.560
		284	25	40/60	25/75 (vol%)	531.18	77.62%	0	3.941
		284 (5X)	5	40/60	25/75 (vol%)	106.24	77.62%	0	47.126
		284 (25X)	1	40/60	25/75 (vol%)	21.25	77.62%	0	71.757
	PIT	285	75	40/60	25/75 (vol%)	531.18	77.78%	0	3.941
		285 (5X)	15	40/60	25/75 (vol%)	106.24	77.78%	0	7.061
		285 (25X)	3	40/60	25/75 (vol%)	21.25	77.78%	0	37.110

Transcutol HP		286	50	40/60	25/75 (vol%)	531.18	77.03%	0	4.269
		286 (5X)	10	40/60	25/75 (vol%)	106.24	77.03%	0	40.394
		286 (25X)	2	40/60	25/75 (vol%)	21.25	77.03%	0	43.350
		287	35	40/60	25/75 (vol%)	531.18	77.75%	0	4.105
		287 (5x)	7	40/60	25/75 (vol%)	106.24	77.75%	0	63.711
		287 (25x)	1.4	40/60	25/75 (vol%)	21.25	77.75%	0	63.383
		288	25	40/60	25/75 (vol%)	531.18	76.95%	0	3.941
		288 (5x)	5	40/60	25/75 (vol%)	106.24	76.95%	0	40.066
		288 (25x)	1	40/60	25/75 (vol%)	21.25	76.95%	0	85.386
	Solvent Displacement	289	50	40/60	25/75 (vol%)	531.18	78.23%	50	0.150
		289 (5x)	10	40/60	25/75 (vol%)	106.24	78.23%	0	16.090
		289 (25x)	2	40/60	25/75 (vol%)	21.25	78.23%	50	50.075
		290	50	50/50	25/75 (vol%)	531.18	77.56%	0	0.000
		290 (5x)	10	50/50	25/75 (vol%)	106.24	77.56%	50	6.466
		290 (25x)	2	50/50	25/75 (vol%)	21.25	77.56%	0	81.805
		291	50	65/35	25/75 (vol%)	531.18	78.28%	0	0.150
		291 (5x)	10	65/35	25/75 (vol%)	106.24	78.28%	0	15.489
		291 (25x)	2	65/35	25/75 (vol%)	21.25	78.28%	0	73.383
		292	50	70/30	25/75 (vol%)	531.18	77.77%	100	0.150
		292 (5x)	10	70/30	25/75 (vol%)	106.24	77.77%	0	3.008
		292 (25x)	2	70/30	25/75 (vol%)	21.25	77.77%	50	73.684
	PIT	293	50	40/60	25/75 (vol%)	531.18	79.06%	50	0.301
		293 (5x)	10	40/60	25/75 (vol%)	106.24	79.06%	50	7.669
		293 (25x)	2	40/60	25/75 (vol%)	21.25	79.06%	100	21.203
		294	50	50/50	25/75 (vol%)	531.18	78.53%	0	0.451
		294 (5x)	10	50/50	25/75 (vol%)	106.24	78.53%	100	5.414
		294 (25x)	2	50/50	25/75 (vol%)	21.25	78.53%	0	43.308
		295	50	65/35	25/75 (vol%)	531.18	79.58%	50	0.000
		295 (5x)	10	65/35	25/75 (vol%)	106.24	79.58%	0	1.053
		295 (25x)	2	65/35	25/75 (vol%)	21.25	79.58%	0	100.000
		296	50	70/30	25/75 (vol%)	531.18	78.02%	0	0.451
		296 (5x)	10	70/30	25/75 (vol%)	106.24	78.02%	100	4.361
		296 (25x)	2	70/30	25/75 (vol%)	21.25	78.02%	100	53.083

Transcutol HP	Solvent Displacement	297	50	35/65	35/65 (vol%)	531.18	73.08%	0	0.819
		297 (5x)	10	35/65	35/65 (vol%)	106.24	73.08%	0	0.702
		297 (25x)	2	35/65	35/65 (vol%)	21.25	73.08%	0	0.819
		298	50	35/65	50/50 (vol%)	531.18	63.77%	0	0.702
		298 (5x)	10	35/65	50/50 (vol%)	106.24	63.77%	0	0.585
		298 (25x)	2	35/65	50/50 (vol%)	21.25	63.77%	0	0.585
		299	50	35/65	65/35 (vol%)	531.18	51.07%	0	0.468
		299 (5x)	10	35/65	65/35 (vol%)	106.24	51.07%	0	0.234
		299 (25x)	2	35/65	65/35 (vol%)	21.25	51.07%	0	2.456
		300	50	35/65	70/30 (vol%)	531.18	43.77%	0	1.053
		300 (5x)	10	35/65	70/30 (vol%)	106.24	43.77%	0	1.520
		300 (25x)	2	35/65	70/30 (vol%)	21.25	43.77%	0	0.234
	PIT	301	75	35/65	35/65 (vol%)	531.18	72.00%	0	0.117
		301 (5x)	15	35/65	35/65 (vol%)	106.24	72.00%	0	0.000
		301 (25x)	3	35/65	35/65 (vol%)	21.25	72.00%	0	0.117
Transcutol HP		302	75	35/65	50/50 (vol%)	531.18	62.71%	0	0.351
		302 (5x)	15	35/65	50/50 (vol%)	106.24	62.71%	0	0.468
		302 (25x)	3	35/65	50/50 (vol%)	21.25	62.71%	0	0.585
	PIT	303	75	35/65	65/35 (vol%)	531.18	52.34%	0	0.468
		303 (5x)	15	35/65	65/35 (vol%)	106.24	52.34%	0	0.234
		303 (25x)	3	35/65	65/35 (vol%)	21.25	52.34%	0	3.626
		304	75	35/65	70/30 (vol%)	531.18	47.50%	0	0.117
		304 (5x)	15	35/65	70/30 (vol%)	106.24	47.50%	0	1.871
		304 (25x)	3	35/65	70/30 (vol%)	21.25	47.50%	0	0.000

Table S3: Global Analysis Pearson's Coefficients.

	Solvent logP Value	Method	Surfactant Molarity	Surfactant Ratio	PBS:Organic Ratio	Hexanol Added	Dilution Factor	Partition Coefficient	Yeast Viability	HeLa Viability
Solvent logP Value	1.000000000	-0.01776019	-0.073419578	0.32202568	0.004050991	-0.014546722	-0.033367217	-0.56868866	0.29760945	0.27015889
Method	-0.017760191	1.00000000	-0.058026264	-0.13666403	0.002820465	-0.341068387	-0.018301564	0.02224677	0.04479591	0.04680279
Surfactant Molarity	-0.073419578	-0.05802626	1.000000000	-0.01297047	0.003183276	0.570945827	-0.666198392	-0.03790646	-0.15130176	-0.29147872
Surfactant Ratio	0.322025681	-0.13666403	-0.012970467	1.00000000	0.073892120	-0.017548233	-0.024765713	-0.11647770	0.19988994	0.08764934
PBS:Organic Ratio	0.004050991	0.002820465	0.003183276	0.07389212	1.000000000	-0.085071569	0.0049367	-0.041464519	-0.06645275	-0.12527662
Hexanol Added	-0.014546722	-0.341068387	0.570945827	-0.01754823	-0.085071569	1.000000000	-0.38726520	-0.007424239	-0.15624660	-0.25896464
Dilution Factor	-0.033367217	-0.018301564	-0.666198392	-0.02476571	0.004936770	-0.387265205	1.000000000	0.01279444	0.12941259	0.23233448
Partition Coefficient	0.568688662	0.02224677	-0.037906463	-0.11647770	-0.041464519	-0.007424239	0.012794438	1.00000000	-0.30160008	-0.30943630
Yeast Viability	0.297418148	0.044280238	-0.150896463	0.19988994	-0.066452751	-0.156246603	0.12941259	-0.301600082	1.00000000	0.60615079
HeLa Viability	0.270158894	0.046892795	-0.291478721	0.08764934	-0.125276616	-0.258964639	0.23233448	-0.309436305	0.60615079	1.0000000

Table S4. Pearson's correlation coefficients by solvent and preparation method

**Iso-octane Solvent Displacement**							
	Surfactant Molarity	Surfactant Ratio	PBS:Organic Ratio	Dilution Factor	Partition Coefficient	Yeast Viability	HeLa Viability
Surfactant Molarity	1.000000000	0.01678467	-0.008944018	-0.723955866	-0.076455571	-0.06495103	-0.25301773
Surfactant Ratio	0.016784666	1.00000000	0.564847837	0.031378508	0.683201041	-0.06702885	-0.39656531
PBS:Organic Ratio	-0.008944018	0.56484784	1.000000000	-0.028490770	0.908569953	-0.34061018	-0.21362622
Dilution Factor	-0.723955866	0.03137851	-0.028490770	1.000000000	-0.001460761	0.12004938	0.38851710
Partition Coefficient	-0.076455571	0.68320104	0.908569953	-0.001460761	1.000000000	-0.28358631	-0.32467996
Yeast Viability	-0.064951033	-0.06702885	-0.340610178	0.120049380	-0.283586307	1.00000000	0.06415459
HeLa Viability	-0.253017726	-0.39656531	-0.213626223	0.388517096	-0.324679964	0.06415459	1.00000000
**Iso-octane Phase Inversion Temperature**							
	Surfactant Molarity	Surfactant Ratio	PBS:Organic Ratio	Dilution Factor	Partition Coefficient	Yeast Viability	HeLa Viability
Surfactant Molarity	1.00000000	-0.01896372	-0.07744022	-0.73205180	0.1663460	-0.4893342	-0.1727304
Surfactant Ratio	-0.01896372	1.00000000	-0.30412421	-0.01457162	-0.2676404	0.4077397	-0.1959913
PBS:Organic Ratio	-0.07744022	-0.30412421	1.00000000	-0.02849077	0.5300216	-0.3687938	-0.2403418
Dilution Factor	-0.73205180	-0.01457162	-0.02849077	1.00000000	-0.1140620	0.4978587	0.3187039
Partition Coefficient	0.16634595	-0.26764040	0.53002155	-0.11406198	1.0000000	-0.2815860	-0.1083648
Yeast Viability	-0.48933425	0.40773967	-0.36879379	0.49785866	-0.2815860	1.0000000	0.1215769
HeLa Viability	-0.17273041	-0.19599128	-0.24034183	0.31870385	-0.1083648	0.1215769	1.0000000
**Captex 355 Solvent Displacement**							
	Surfactant Molarity	Surfactant Ratio	PBS:Organic Ratio	Dilution Factor	Partition Coefficient	Yeast Viability	HeLa Viability
Surfactant Molarity	1.0000000000	0.003486162	0.0007105408	-0.72395587	-0.107243376	-0.613399902	-0.45044965
Surfactant Ratio	0.0034861617	1.000000000	-0.0942602974	-0.01967016	0.508345113	0.040655146	-0.08816047
PBS:Organic Ratio	0.0007105408	-0.094260297	1.0000000000	-0.11280492	0.190009725	-0.348630905	0.01467710
Dilution Factor	-0.7239558657	-0.019670158	-0.1128049202	1.00000000	0.087466201	0.799129514	0.71659405
Partition Coefficient	-0.1072433757	0.508345113	0.1900097254	0.08746620	1.000000000	0.004172665	0.02244060
Yeast Viability	-0.6133999017	0.040655146	-0.3486309050	0.79912951	0.004172665	1.000000000	0.55424825
HeLa Viability	-0.4504496465	-0.088160469	0.0146770952	0.71659405	0.022440603	0.554248247	1.00000000

Captex 355 Phase Inversion Temperature							
	Surfactant Molarity	Surfactant Ratio	PBS:Organic Ratio	Dilution Factor	Partition Coefficient	Yeast Viability	HeLa Viability
Surfactant Molarity	1.0000000	-0.10394817	-0.21364873	-0.63644264	-0.33417327	-0.5896233	-0.39050122
Surfactant Ratio	-0.1039482	1.00000000	-0.09426030	-0.01967016	0.65201055	0.2492051	-0.01156849
PBS:Organic Ratio	-0.2136487	-0.09426030	1.00000000	-0.11280492	0.45597815	-0.2903121	-0.08484432
Dilution Factor	-0.6364426	-0.01967016	-0.11280492	1.00000000	0.07229237	0.6504543	0.71101250
Partition Coefficient	-0.3341733	0.65201055	0.45597815	0.07229237	1.00000000	0.2630251	-0.06203156
Yeast Viability	-0.5896233	0.24920506	-0.29031213	0.65045426	0.26302515	1.0000000	0.56030736
HeLa Viability	-0.3905012	-0.01156849	-0.08484432	0.71101250	-0.06203156	0.5603074	1.00000000

Capmul MCM Solvent Displacement							
	Surfactant Molarity	Surfactant Ratio	PBS:Organic Ratio	Dilution Factor	Partition Coefficient	Yeast Viability	HeLa Viability
Surfactant Molarity	1.00000000	-0.05389185	-0.17093013	-0.64315252	0.3644613	-0.02478937	0.50539454
Surfactant Ratio	-0.05389185	1.00000000	-0.30412421	-0.01457162	0.1821324	-0.20015302	-0.08510627
PBS:Organic Ratio	-0.17093013	-0.30412421	1.00000000	-0.02849077	-0.6404720	0.39090071	-0.24935011
Dilution Factor	-0.64315252	-0.01457162	-0.02849077	1.00000000	-0.0989737	-0.03706247	-0.12405383
Partition Coefficient	0.36446128	0.18213237	-0.64047196	-0.09897370	1.0000000	-0.27983896	0.38824333
Yeast Viability	-0.02478937	-0.20015302	0.39090071	-0.03706247	-0.2798390	1.00000000	-0.23552786
HeLa Viability	0.50539454	-0.08510627	-0.24935011	-0.12405383	0.3882433	-0.23552786	1.00000000

Capmul MCM Phase Inversion Temperature							
	Surfactant Molarity	Surfactant Ratio	PBS:Organic Ratio	Dilution Factor	Partition Coefficient	Yeast Viability	HeLa Viability
Surfactant Molarity	1.00000000	-0.01272073	-0.13232329	-0.69715174	-0.01999339	0.3924126	x
Surfactant Ratio	-0.01272073	1.00000000	-0.39742810	-0.01967016	0.21667143	0.1643730	x
PBS:Organic Ratio	-0.13232329	-0.39742810	1.00000000	-0.02849077	-0.36385056	0.1243130	x
Dilution Factor	-0.69715174	-0.01967016	-0.02849077	1.00000000	0.11370926	-0.3637436	x
Partition Coefficient	-0.01999339	0.21667143	-0.36385056	0.11370926	1.00000000	-0.3076379	x
Yeast Viability	0.39241258	0.16437305	0.12431304	-0.36374356	-0.30763791	1.0000000	x
HeLa Viability	x	x	x	x	x	x	x

Lauroglycol 90 Solvent Displacement							
	Surfactant Molarity	Surfactant Ratio	PBS:Organic Ratio	Dilution Factor	Partition Coefficient	Yeast Viability	HeLa Viability
Surfactant Molarity	1.000000000	0.01678467	-0.008944018	-0.72395587	-0.02233419	-0.3354786	-0.86944336
Surfactant Ratio	0.016784666	1.00000000	0.564847837	0.03137851	0.58212008	0.3298399	0.14976806
PBS:Organic Ratio	-0.008944018	0.56484784	1.000000000	-0.02849077	0.98012753	0.3369058	0.08307501
Dilution Factor	-0.723955866	0.03137851	-0.028490770	1.00000000	-0.02792574	0.2018721	0.80572977
Partition Coefficient	-0.022334191	0.58212008	0.980127526	-0.02792574	1.00000000	0.2516437	0.07022550
Yeast Viability	-0.335478610	0.32983987	0.336905802	0.20187212	0.25164371	1.0000000	0.46330931
HeLa Viability	-0.869443361	0.14976806	0.083075007	0.80572977	0.07022550	0.4633093	1.00000000
**Lauroglycol 90 Phase Inversion Temperature**							
	Surfactant Molarity	Surfactant Ratio	PBS:Organic Ratio	Dilution Factor	Partition Coefficient	Yeast Viability	HeLa Viability
Surfactant Molarity	1.000000000	0.01461402	-0.008944018	-0.72395587	0.003242925	-0.3274657	-0.8808388
Surfactant Ratio	0.014614017	1.00000000	0.222339689	0.01361190	0.420652852	0.3162989	0.1338753
PBS:Organic Ratio	-0.008944018	0.22233969	1.000000000	-0.02849077	0.319650131	0.1998136	0.1474238
Dilution Factor	-0.723955866	0.01361190	-0.028490770	1.00000000	0.031722696	0.4471821	0.7170625
Partition Coefficient	0.003242925	0.42065285	0.319650131	0.03172270	1.000000000	0.4719023	0.2468372
Yeast Viability	-0.327465742	0.31629891	0.199813554	0.44718212	0.471902304	1.0000000	0.6008062
HeLa Viability	-0.880838833	0.13387528	0.147423826	0.71706252	0.246837247	0.6008062	1.0000000
**Lauroglycol FCC Solvent Displacement**							
	Surfactant Molarity	Surfactant Ratio	PBS:Organic Ratio	Dilution Factor	Partition Coefficient	Yeast Viability	HeLa Viability
Surfactant Molarity	1.00000000	-0.25950231	-0.17093013	-0.64315252	-0.08150099	0.09994171	0.31383596
Surfactant Ratio	-0.25950231	1.00000000	0.56484784	0.03137851	-0.18051212	-0.02897109	0.01177917
PBS:Organic Ratio	-0.17093013	0.56484784	1.00000000	-0.02849077	-0.03618186	-0.01015775	0.33908155
Dilution Factor	-0.08150099	0.03137851	-0.02849077	1.00000000	0.03242962	-0.05330088	-0.22416654
Partition Coefficient	0.09994171	-0.18051212	-0.03618186	0.03242962	1.00000000	-0.02230988	-0.44064393
Yeast Viability	0.31383596	-0.02897109	-0.01015775	-0.05330088	-0.02230988	1.00000000	0.53395256
HeLa Viability	0.31383596	0.01177917	0.33908155	-0.22416654	-0.44064393	0.53395256	1.00000000

**Lauroglycol FCC Phase Inversion Temperature**							
	Surfactant Molarity	Surfactant Ratio	PBS:Organic Ratio	Dilution Factor	Partition Coefficient	Yeast Viability	HeLa Viability
Surfactant Molarity	1.00000000	-0.01808493	-0.17093013	-0.64315252	-0.01139017	0.04200189	-0.24101508
Surfactant Ratio	-0.01808493	1.00000000	-0.39742810	-0.01967016	-0.45890783	-0.27244359	-0.48045634
PBS:Organic Ratio	-0.17093013	-0.39742810	1.00000000	-0.02849077	0.93253737	-0.05365991	0.37599625
Dilution Factor	-0.64315252	-0.01967016	-0.02849077	1.00000000	-0.13779902	0.06771436	0.02552305
Partition Coefficient	-0.01139017	-0.45890783	0.93253737	-0.13779902	1.00000000	0.09713609	0.52019948
Yeast Viability	0.04200189	-0.27244359	-0.05365991	0.06771436	0.09713609	1.00000000	0.26918643
HeLa Viability	-0.24101508	-0.48045634	0.37599625	0.02552305	0.52019948	0.26918643	1.00000000
**Labrafac PG Solvent Displacement**							
	Surfactant Molarity	Surfactant Ratio	PBS:Organic Ratio	Dilution Factor	Partition Coefficient	Yeast Viability	HeLa Viability
Surfactant Molarity	1.00000000	-0.19212556	-0.13232329	-0.69715174	-0.07754654	0.13703904	-0.6294294
Surfactant Ratio	-0.19212556	1.00000000	0.56484784	0.03137851	0.17075332	-0.30975536	0.1212566
PBS:Organic Ratio	-0.13232329	0.56484784	1.00000000	-0.02849077	-0.35388804	-0.35323681	-0.1268151
Dilution Factor	-0.69715174	0.03137851	-0.02849077	1.00000000	0.04469208	0.30179374	0.9069463
Partition Coefficient	-0.07754654	0.17075332	-0.35388804	0.04469208	1.00000000	0.07164182	0.2286659
Yeast Viability	0.13703904	-0.30975536	-0.35323681	0.30179374	0.07164182	1.00000000	0.3384485
HeLa Viability	-0.62942940	0.12125658	-0.12681508	0.90694626	0.22866587	0.33844845	1.0000000
**Labrafac PG Phase Inversion Temperature**							
	Surfactant Molarity	Surfactant Ratio	PBS:Organic Ratio	Dilution Factor	Partition Coefficient	Yeast Viability	HeLa Viability
Surfactant Molarity	1.0000000	-0.25950220	-0.17093013	-0.643152516	-0.126205218	-0.13466977	-0.37269348
Surfactant Ratio	-0.2595022	1.00000000	0.56484778	0.031378451	0.622476486	-0.15070230	-0.21297507
PBS:Organic Ratio	-0.1709301	0.56484778	1.00000000	-0.028490768	0.069825607	-0.01050105	-0.07140894
Dilution Factor	-0.6431525	0.03137845	-0.02849077	1.000000000	0.002855889	0.34204364	0.37061294
Partition Coefficient	-0.1262052	0.62247649	0.06982561	0.002855889	1.000000000	-0.21478220	-0.22045214
Yeast Viability	-0.1346698	-0.15070230	-0.01050105	0.342043636	-0.214782199	1.00000000	0.22756502
HeLa Viability	-0.3726935	-0.21297507	-0.07140894	0.370612939	-0.220452139	0.22756502	1.00000000

Transcutol HP Solvent Displacement							
	Surfactant Molarity	Surfactant Ratio	PBS:Organic Ratio	Dilution Factor	Partition Coefficient	Yeast Viability	HeLa Viability
Surfactant Molarity	1.00000000	-0.01896382	-0.07744022	-0.732051801	0.088762139	0.17109572	-0.47317639
Surfactant Ratio	-0.01896382	1.00000000	-0.30412393	-0.014571681	0.321919608	0.23383016	0.13901606
PBS:Organic Ratio	-0.07744022	-0.30412393	1.00000000	-0.028490768	-0.996835971	-0.24435877	-0.34778235
Dilution Factor	-0.73205180	-0.01457168	-0.02849077	1.000000000	0.008103975	-0.06114076	0.68207487
Partition Coefficient	0.08876214	0.32191961	-0.99683597	0.008103975	1.000000000	0.25129269	0.35646928
Yeast Viability	0.17109572	0.23383016	-0.24435877	-0.061140764	0.251292687	1.00000000	0.05892067
HeLa Viability	-0.47317639	0.13901606	-0.34778235	0.682074870	0.356469285	0.05892067	1.00000000
**Transcutol HP Phase Inversion Temperature**							
	Surfactant Molarity	Surfactant Ratio	PBS:Organic Ratio	Dilution Factor	Partition Coefficient	Yeast Viability	HeLa Viability
Surfactant Molarity	1.00000000	-0.08075619	0.04849743	-0.71831287	-0.06646160	-0.21597346	-0.43920262
Surfactant Ratio	-0.08075619	1.00000000	-0.30412393	-0.01457168	0.35846448	0.57418528	-0.04410743
PBS:Organic Ratio	0.04849743	-0.30412393	1.00000000	-0.02849077	-0.98882978	-0.24672069	-0.30584436
Dilution Factor	-0.71831287	-0.01457168	-0.02849077	1.00000000	0.01842841	0.16258417	0.59251003
Partition Coefficient	-0.06646160	0.35846448	-0.98882978	0.01842841	1.00000000	0.29151220	0.34648023
Yeast Viability	-0.21597346	0.57418528	-0.24672069	0.16258417	0.29151220	1.00000000	0.08960581
HeLa Viability	-0.43920262	-0.04410743	-0.30584436	0.59251003	0.34648023	0.08960581	1.00000000

Table S5: Self-Nanoemulsion RFP formulation compositions and partition coefficients.

	Sample Number	Water Loading	Surfactant Molarity (mM)	Surfactant Ratio (10MAG/LDAO)	Concentration of RFP injected (uM)	Partition Coefficient
Lauroglycol 90	630	15	75	60/40	0.397	5.22
	631	20	75	60/40	0.397	11.57
Iso-octane	664	15	75	70/30	0.397	55.16
	665	20	75	70/30	0.397	68.48
	666	15	75	60/40	0.397	0.13
	667	20	75	60/40	0.397	59.26
	668	15	75	65/35	0.397	63.85
	669	20	75	65/35	0.397	64.09
Capmul MCM	700	15	75	70/30	0.397	26.94
	701	20	75	70/30	0.397	48.39
	702	15	75	60/40	0.397	53.74
	703	20	75	60/40	0.397	61.02
	704	15	75	65/35	0.397	52.18
	705	20	75	65/35	0.397	58.26

Table S6: Solvent-only or solvent plus Propidium Iodide treatment of S. cerevisiae and HeLa cells

		HeLa Cytotoxicity Data		S. cerevisiae Permeability
**Solvent**	**Dilution**	**Raw Signal**	**Percentage Viability**	**Percentage Permeability**
PBS	None	395	100.00	7.95
CTAB	None	34	0.00	99.8
Transcutol HP	No Propidium Iodide	47	3.60	99.35
	Propidium Iodide	40	1.66	97.9
	1:10 Dilution PI	45	3.05	96.65
	1:100 Dilution PI	44	2.77	97.85

Capmul	No Propidium Iodide	30	0.00	34.2
	Propidium Iodide	40	1.66	22.75
	1:10 Dilution PI	46	3.32	23.3
	1:100 Dilution PI	38	1.11	21.9
Capryol 90	No Propidium Iodide	36	0.55	67.7
	Propidium Iodide	35	0.28	78.95
	1:10 Dilution PI	36	0.55	68
	1:100 Dilution PI	37	0.83	80.6
IsoOctane	No Propidium Iodide	513	100.00	1.8
	Propidium Iodide	348	86.98	2.65
	1:10 Dilution PI	318	78.67	1.85
	1:100 Dilution PI	335	83.38	4.15
Captex	No Propidium Iodide	340	84.76	2.6
	Propidium Iodide	319	78.95	3.35
	1:10 Dilution PI	317	78.39	4.5
	1:100 Dilution PI	308	75.90	5.15
Lauroglycol 90	No Propidium Iodide	285	69.53	74
	Propidium Iodide	383	96.68	29.85
	1:10 Dilution PI	252	60.39	52
	1:100 Dilution PI	217	60.39	63.3
Lauroglycol FCC	No Propidium Iodide	428	100.00	39.25
	Propidium Iodide	254	60.94	44.65
	1:10 Dilution PI	413	100.00	13.35
	1:100 Dilution PI	226	53.19	25.9
Labrafac PG	No Propidium Iodide	634	100.00	4.5
	Propidium Iodide	432	100.00	2.95
	1:10 Dilution PI	487	100.00	5.05
	1:100 Dilution PI	359	90.03	4.2
